# Structural basis for disruption of claudin assembly in tight junctions by an enterotoxin

**DOI:** 10.1038/srep33632

**Published:** 2016-09-20

**Authors:** Takehiro Shinoda, Naoko Shinya, Kaori Ito, Noboru Ohsawa, Takaho Terada, Kunio Hirata, Yoshiaki Kawano, Masaki Yamamoto, Tomomi Kimura-Someya, Shigeyuki Yokoyama, Mikako Shirouzu

**Affiliations:** 1 RIKEN Systems and Structural Biology Center, 1-7-22 Suehiro-cho, Tsurumi-ku, Yokohama 230-0045, Japan; 2 Division of Structural and Synthetic Biology, RIKEN Center for Life Science Technologies, 1-7-22 Suehiro-cho, Tsurumi-ku, Yokohama 230-0045, Japan; 3 RIKEN Structural Biology Laboratory, 1-7-22 Suehiro-cho, Tsurumi-ku, Yokohama 230-0045, Japan; 4 RIKEN SPring-8 Center, 1-1-1, Kouto, Sayo-cho, Sayo-gun, Hyogo 679-5148, Japan

## Abstract

The food-poisoning bacterium *Clostridium perfringens* produces an enterotoxin (~35 kDa) that specifically targets human claudin-4, among the 26 human claudin proteins, and causes diarrhea by fluid accumulation in the intestinal cavity. The C-terminal domain of the *Clostridium perfringens* enterotoxin (C-CPE, ~15 kDa) binds tightly to claudin-4, and disrupts the intestinal tight junction barriers. In this study, we determined the 3.5-Å resolution crystal structure of the cell-free synthesized human claudin-4•C-CPE complex, which is significantly different from the structure of the off-target complex of an engineered C-CPE with mouse claudin-19. The claudin-4•C-CPE complex structure demonstrated the mechanism underlying claudin assembly disruption. A comparison of the present C-CPE-bound structure of claudin-4 with the enterotoxin-free claudin-15 structure revealed sophisticated C-CPE-induced conformation changes of the extracellular segments, induced on the foundation of the rigid four-transmembrane-helix bundle structure. These conformation changes provide a mechanistic model for the disruption of the lateral assembly of claudin molecules. Furthermore, the present novel structural mechanism for selecting a specific member of the claudin family can be used as the foundation to develop novel medically important technologies to selectively regulate the tight junctions formed by claudin family members in different organs.

In vertebrate epithelial cell sheets that separate organs, cell-cell adhesion structures designated as tight junctions serve as almost impermeable fluid barriers. Claudins are ~23-kDa four-α-helical transmembrane proteins present in tight junctions, where they assemble into “tight-junction strands” to seal the intercellular space^1^,^2^. Mammals have 27 genes encoding claudin-family members, which are expressed tissue-specifically^3^. Claudin gene knockouts or mutations cause dysfunctions in the tight junction, and seriously affect tissue functions. In fact, the claudin-1 and claudin-5 knockout mice die postnatally^4^,^5^. Furthermore, deficiencies in claudin functions cause human diseases, such as neonatal sclerosing cholangitis associated with ichthyosis (claudin-1)^6^, deafness (claudin-11 and claudin-14)^7^,^8^, and familial hypomagnesemia with hypercalciuria and nephrocalcinosis (claudin-16 and claudin-19)^9^,^10^. The bacterium *Clostridium perfringens* causes food poisoning by producing the *C. perfringens* enterotoxin (CPE), which binds tightly to claudin-4 and less strongly to claudin-3^11^. CPE consists of the N-terminal cytotoxic β-pore-forming domain and the C-terminal claudin-binding domain (C-CPE). The C-CPE domain disrupts the barrier and causes the endocytic internalization of tight junctions^12^. However, the mechanism by which the tight junction becomes disrupted has remained unknown.

Most of the claudin family members, including claudin-4, were quite difficult to prepare by cellular expression^13^. However, in 2014, a mutant of mouse claudin-15 was successfully prepared by cellular expression, and its crystal structure was determined^13^. On the basis of the crystal packing, a model of the *cis* interaction of claudin molecules in the tight-junction strand was proposed^13^. More recently, a mutant of mouse claudin-19 was prepared in a similar manner, and the crystal structure of its complex with the S313A mutant of C-CPE, C-CPE_203-319_S313A, which binds claudin-19 with higher affinity, was solved^14^. On the basis of the complex structure, a mechanism of tight-junction disruption was proposed^14^. However, claudin-19 is not the authentic binding target of C-CPE, and the artificial effects of the S313A mutation on the interaction with C-CPE could not be determined.

Recently, we succeeded in the preparation of human claudin-4 with a deletion of the intracellular C-terminal 26 residues (residues 1–183), by a novel cell-free protein synthesis method using an *Escherichia coli* cell extract^15^, and observed its C-CPE binding with the dissociation constant of 3.4 nM^16^. This success is ascribed to the use of the cell-free method, rather than cellular expression. The claudin-4•C-CPE complex was crystallized and diffracted to about 4 Å-resolution. We also prepared an N-terminal fusion of the human claudin-4 protein with T4 phage lysozyme (T4L, residues 2–162) through a glycine residue, by the cell-free method, and its co-crystal with C-CPE diffracted up to 3.5-Å resolution^16^.

In this study, we determined the 3.5-Å resolution crystal structure of the cell-free synthesized T4L-fused human claudin-4•C-CPE complex (designated hereafter as the human claudin-4•C-CPE complex). The crystal structure revealed the mechanism underlying the disruption of the claudin-4 assembly by C-CPE. The mode of the “on-target” interactions of C-CPE with claudin-4 is appreciably different from that of the “off-target” interactions of claudin-19 with the S313A mutant of C-CPE. This structural information will facilitate the development of novel therapeutics.

## Results

**Figure 1:**
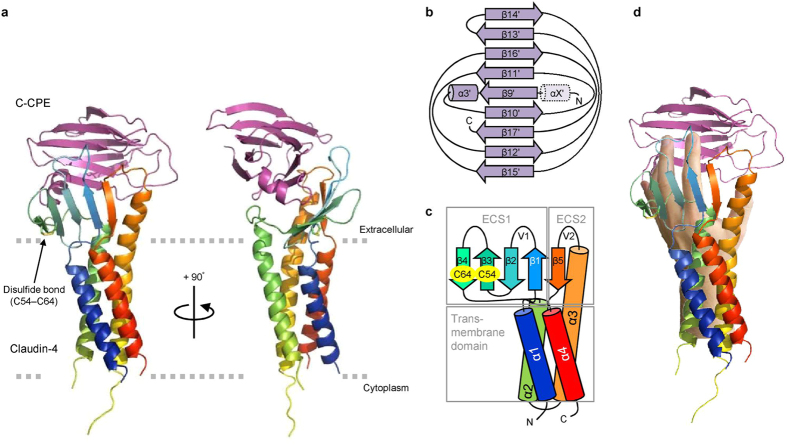
Crystal structure of the complex of human claudin-4 and C-CPE. (**a**) The crystal structure of the claudin-4•C-CPE complex. Claudin-4 is colored in rainbow. C-CPE is colored purple. (**b,c**) Secondary structures of C-CPE and human claudin-4, respectively. The α helices and β strands of C-CPE are numbered in accordance to the previously reported full-length structure^17^, except for αX′ in complexes II and IV. (**d**) The crystal structure of the human claudin-4•C-CPE complex, superimposed on an author’s left hand.

The crystal structure of the complex was solved at 3.5-Å resolution (Fig. 1a, Supplementary Table S1, Supplementary Figs S1 and S2a–d). The asymmetric unit, with the space group *P*4_3_, contains four non-crystallographic symmetry-related complexes (I–IV) (Supplementary Fig. S3a). These complexes essentially adopt the same overall structures (Supplementary Figs S3c and S4a–c), except for the presence of helix αX′ of C-CPE in complexes II and IV (Fig. 1b and Supplementary Figs S3a and S5a). Helix αX′ of C-CPE was not reported in the previous structure of the full-length CPE (PDB: 3AM2)^17^. Complexes II and IV are adjacent to each other in an upside-down manner (Supplementary Fig. S3a). In the upside-down arrangement of these complexes, the T4L tag of one complex interacts with the C-CPE of the other and probably thereby generates helix αX′, and vice versa (Supplementary Fig. S3a). The complex I structure is described hereafter, unless otherwise noted (Supplementary Fig. S3a–c).

**Figure 2:**
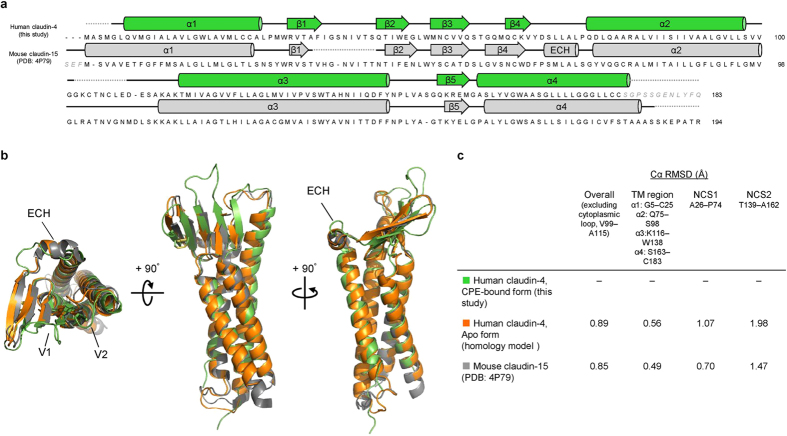
Structural comparison between human claudin-4 in the C-CPE-bound form and mouse claudin-15 in the apo form. (**a**) Alignment of the primary and secondary structures of the C-CPE-bound form (complex I) of human claudin-4 (upper) and the apo form of mouse claudin-15 (PDB: 4P79)^13^ (lower). The primary structures were aligned with Clustal Omega. (**b**) Superimposition of the tertiary structures of the C-CPE-bound form of human claudin-4 (green), the homology-modeled apo form of human claudin-4 (orange), and the apo form of mouse claudin-15 (gray). (**c**) Summary of the r. m. s. d. of the Cα atoms between the structures.

**Figure 3:**
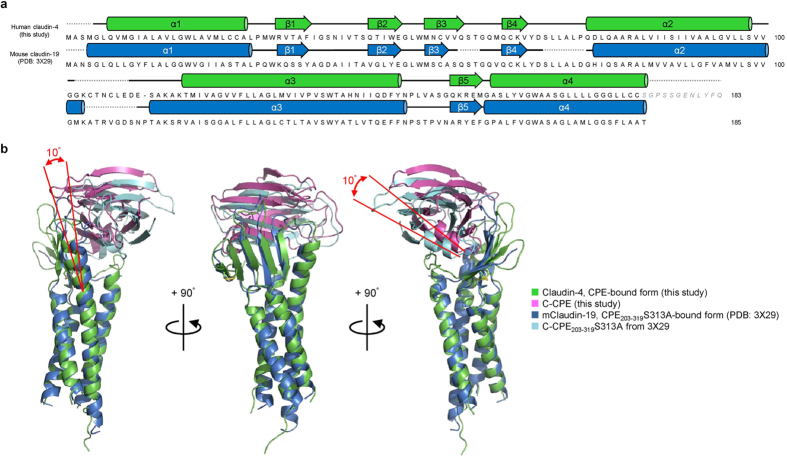
Structural differences between the human claudin-4•C-CPE complex and the mouse claudin-19•C-CPE mutant complex. (**a**) Alignment of the primary and secondary structures of the C-CPE-bound form (complex I) of human claudin-4 (upper) and the C-CPE mutant-bound form of mouse claudin-19 (PDB: 3 X 29)^14^ (lower). The primary structures were aligned with Clustal Omega (http://www.ebi.ac.uk/Tools/msa/clustalo/). (**b**) Superimposition of the tertiary structures of the present human claudin-4•C-CPE complex and the mouse claudin-19•C-CPE mutant complex.

**Figure 4:**
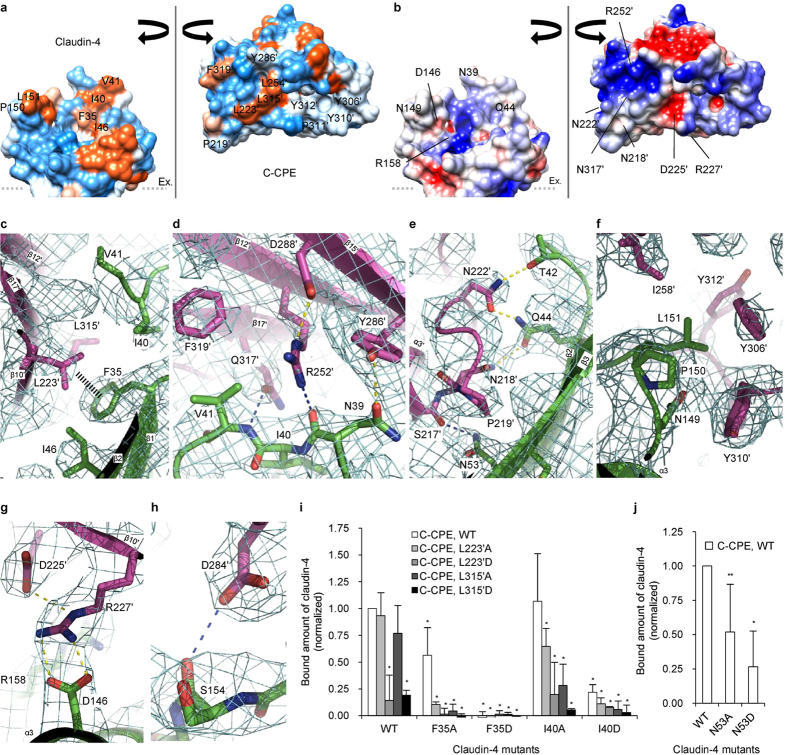
The CPE-binding site of human claudin-4. (**a**) The hydrophobic potential, color-coded from the hydrophobic area (orange) to the hydrophilic area (light-blue), on the surfaces of claudin-4 and C-CPE with their interfaces presented toward the front. (**b**) The electrostatic potential, color-coded from −2 *kT/e* (red) to +2 *kT/e* (blue), on the claudin-4 and C-CPE surfaces presented as in (**a**). (**c–h**) The CPE-binding sites of ECS1 (**c–e**) and ECS2 (**f–h**). Claudin-4 and C-CPE are represented in green and purple, respectively. The yellow and sky-blue dashed lines indicate the side-chain•side-chain and the main-chain•side-chain hydrophilic interactions, respectively. The black dashed line indicates a van der Waals interaction. The electron density maps, 2*F*o-*F*c, were contoured at 1 σ and are colored light-blue. (**i,j**) The pull-down assay of claudin-4 with C-CPE. Various mutations of the claudin-4 ECS1 and C-CPE residues involved in the hydrophobic and hydrophilic interactions in (**c,e**), respectively, with means ± S.E. (error bar); *n* = 4, *p < 0.05, **p < 0.1 to the value between the wild-type (WT) claudin-4 and C-CPE.

The structure of C-CPE in complex with claudin-4 is nearly the same as that of the CPE protein itself^17^, indicating the absence of a conformational change in C-CPE upon complex formation (Supplementary Fig. S5a,b). The transmembrane parts of the four helices of claudin-4 superimposed well on those of mouse claudin-15 alone^13^ and mouse claudin-19 in complex with the S313A mutant C-CPE^14^ (Figs 2a–c and 3a,b). The two extracellular segments, ECS1 and ECS2, of claudin-4 both interact with C-CPE, and the complex resembles a left hand grasping the ellipsoidal C-CPE (Fig. 1c,d). The ECS1 and ECS2 regions of claudin-4 undergo conformation changes upon complex formation, as described below (Fig. 4).

The ECS1 region of claudin-4 is intimately involved in the association with C-CPE. This interaction mode of ECS1 is likely to be conserved in the S313A C-CPE complex of claudin-19^14^. Therefore, the hydrophobic residues in the protein-protein interface (Fig. 4a–c) were mutated (Fig. 4i). First, the F35D mutation of claudin-4 abolished the CPE-binding ability, and thus F35 is required for the association. F35 and L223′ form the closest intermolecular contact within this hydrophobic interface (Fig. 4c). Correspondingly, the L223′D mutation of C-CPE drastically impaired the claudin-4-binding ability. The effects of the F35D and L223′D mutations are much larger than those of the F35A and L223′A mutations, respectively, indicating the hydrophobic nature of the interaction of C-CPE with the ECS1 of claudin-4. In addition, ECS1 and C-CPE form extensive hydrophilic interactions neighboring the hydrophobic interface. The ECS1 β1 and β2 strands exhibit good shape complementarities to C-CPE, and interact with three hydrogen bonds, N39–R252′, N39–Y286′, and I40–Q317′ (Fig. 4d). N53 on the ‘ring finger’ (β3) of claudin-4 hydrogen bonds with S217′ (Fig. 4e). Actually, the N53A and N53D mutations decreased the CPE-binding ability of claudin-4 (Fig. 4j). In this context, the deletion of residues 184′–220′ of C-CPE reportedly decreased its affinity for claudin-4^12^.

The claudin-4 ECS2 (the thumb in Fig. 1d) is also involved in the association with C-CPE. In contrast to the ECS1 interactions, this interaction mode is not conserved in the claudin-19 complex^14^. First of all, L151 from the claudin-4 ECS2 is accommodated in the “triple tyrosine pit”, consisting of Y306′, Y310′, and Y312′, in the present complex structure (Fig. 4f), which is consistent with the previous mutagenesis studies^18^. In addition, D146 from the claudin-4 ECS2 domain interacts with R227′ of C-CPE (Fig. 4b,g). A previous mutagenesis analysis of D146 revealed the importance of this residue in CPE binding^19^, and the present study has now identified its interaction partner as R227′. Furthermore, the C-CPE-binding interface of the claudin-4 ECS2 domain involves the hydrogen bond S154–D284′ (Fig. 4h). Notably, CPE binds claudin-4 much more strongly than the other claudins. Among the claudins, the ECS2 domain of claudin-4 exhibits much lower sequence homology than the ECS1 domain (Supplementary Fig. S6, V2 region), and has been discussed as the site that defines the CPE-binding specificity^20^.

We compared our crystal structure of the human claudin-4•C-CPE complex with that of the mouse claudin-19•C-CPE_203-319_S313A complex (PDB: 3 X 29)^14^ (Fig. 3a,b). Although claudin-19 is not a biological target of CPE, it still binds to C-CPE with about 70-fold weaker affinity than claudin-4^14^. We detected several important differences between the two structures. First, the orientation of C-CPE relative to the transmembrane helices is different, by ~10°, between the claudin-4 and claudin-19 complexes (Fig. 3b). The extracellular part of the third TM helix of claudin-4 is bent by ~10° toward the outside, as compared to that of claudin-19, while in contrast, the transmembrane parts of the third TM helices are highly superimposable (Fig. 3b).

Second, twelve interactions between claudin-4 and C-CPE were observed (Fig. 4c–h), but five of them, N39–Y286′, Q44–N218′, Q44–N222′, N53–S217′, and L151–Y306′/Y310′/Y312′ (triple tyrosine pit), are missing in the claudin-19•C-CPE_203-319_S313A complex^14^. Conversely, six C-CPE_203-319_S313A-interacting residues of claudin-19 (D38–K283′, S53–N218′, T153–R227′, T153–S256′, N156–D284′, and Y159–D225′)^14^ are missing among the C-CPE-interacting residues of claudin-4.

The F35 residue in claudin-4 is replaced by Tyr in claudin-19. In addition, L151 in claudin-4, which interacts with the triple tyrosine pit, is replaced by Ser in claudin-19 (Fig. 3a). Therefore, the hydrophobic interaction of C-CPE with claudin-19 is much weaker than that with claudin-4. Furthermore, S38 in the ECS1 of claudin-4 is replaced by Asp in the ECS1 of claudin-19 (Fig. 3a), in which D38 forms a salt bridge with K283′. According to Saitoh *et al*.^14^, the D38A mutation of claudin-19 strengthens the interaction with C-CPE^14^. Therefore, the salt bridging D38–K283′ interaction prevents the claudin-19•C-CPE complex from forming the correct conformation around the ECS1 of claudin-19.

**Figure 5:**
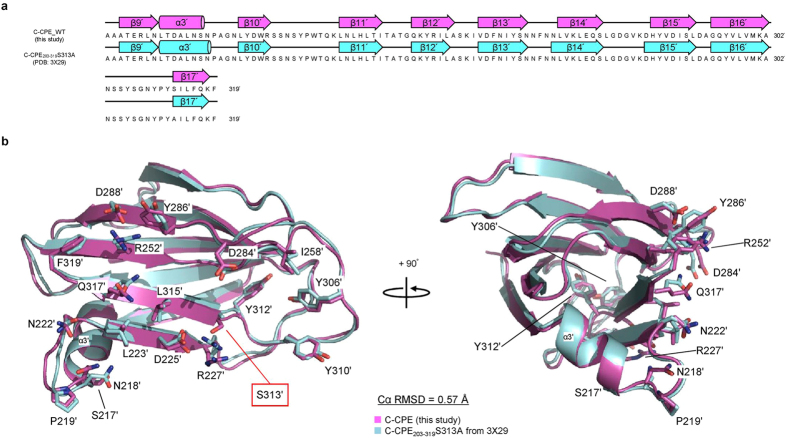
Structural differences between C-CPE_WT and C-CPE_203-319_S313A. (**a**) Alignment of the primary and secondary structures of the C-CPE_WT from the present human claudin-4•C-CPE complex (upper) and the C-CPE_203-319_S313A from the mouse claudin-19•C-CPE mutant (PDB: 3 X 29)^14^ (lower). The primary structures were aligned with Clustal Omega (http://www.ebi.ac.uk/Tools/msa/clustalo/). (**b**) Superimposition of the tertiary structures of the C-CPE_WT (purple) and the C-CPE_203-319_S313A mutant (light blue). The residues in the claudin-4-binding site of C-CPE_WT are shown by stick models.

**Figure 6:**
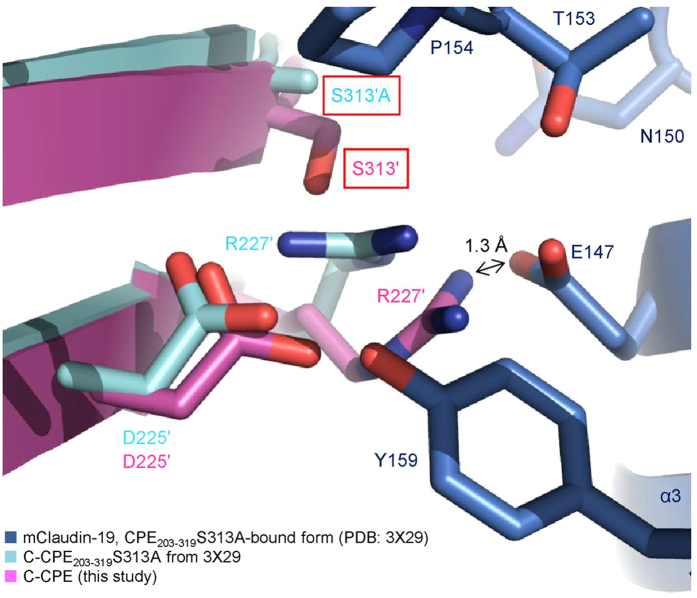
The mutation of S313′ to alanine in C-CPE alleviates the steric hindrance between R227′ and E147 on the α3 helix of claudin-19. The wild-type C-CPE from the present human claudin-4•C-CPE complex (purple) was superimposed onto the C-CPE_203-319_S313A in the mouse claudin-19•C-CPE mutant complex (PDB: 3 X 29)^14^ (light blue). The black double-headed arrow indicates the distance between E147 of mouse claudin-19 (dark blue) and R227′ of the wild-type C-CPE.

Why does C-CPE_203-319_S313A tightly bind to claudin-19? The structures of C-CPE and C-CPE_203-319_S313A are very similar to each other (Fig. 5a,b). In the present structure, the side chain of R227′, near S313′, of C-CPE and that of D146 of claudin-4 face each other (Fig. 4g). However, the side chain of E147 in claudin-19 is longer than that of D146 in claudin-4, by about 1.5 Å. Therefore, when the wild-type C-CPE binds to claudin-19, the E147 side chain is as close as 1.3 Å to the R227′ side chain of C-CPE (Fig. 6). Actually, in the claudin-19•C-CPE_203-319_S313A complex^14^, the side chain of R227′ of C-CPE_203-319_S313A is bent. The mutation of S313′ to alanine provides sufficient space for R227′ to avoid the steric hindrance with E147. Consequently, C-CPE_203-319_S313A can bind tightly to claudin-19.

**Figure 7:**
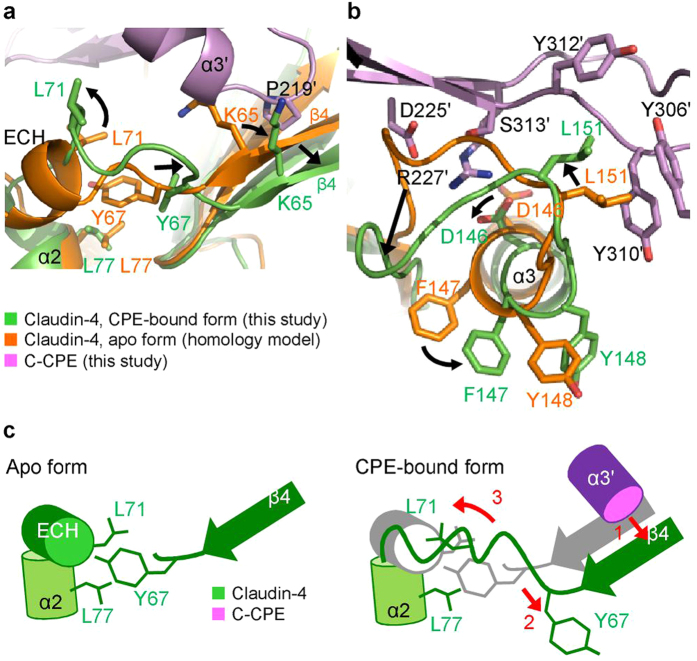
Conformation changes of human claudin-4 by C-CPE-binding. (**a,b**) Superimposition of the CPE-bound form and the homology-modeled apo form of human claudin-4. The black arrows indicate the claudin-4 residue movements induced by C-CPE binding, due to the conformation changes of β4–α2 of ECS1 (**a**) and ECS2 (**b**). **(c)** Schematic representation of the C-CPE-induced conformation changes in β4–α2 of ECS1.

We superimposed a homology model of the human claudin-4 apo form, constructed on the basis of the structure of the mouse claudin-15 apo form^13^, on the present claudin-4•C-CPE complex structure (Fig. 2b,c and Fig. 7a,b). The superimposition revealed that significant conformation changes occur upon CPE binding, in both the ECS1 and ECS2 domains. In the apo form, a short α helix, designated as the extracellular helix (ECH), exists between β4 and α2, whereas this region is unwound and extended in the C-CPE-bound form (Figs 2b and 7a). In the ECS2 region, the loop between the extracellular part of helix α3 and strand β5, designated as the V2 region, assumes different conformations between the two forms (Fig. 7b). In contrast, the other parts of the ECS1 region (β1 and β2) and the transmembrane helix bundle are highly superimposable (Fig. 2b). Consequently, the C-CPE can bind firmly to this rigid foundation (the transmembrane helices and the ECS1 β1 and β2) of claudin-4. The α3′ helix of C-CPE then pushes strand β4 outward by about 2 Å toward the ‘outside of the hand’ (Figs 1b and 7a). Concomitantly, the van der Waals interaction is disrupted between Y67 and L71 on the ECH and L77 on helix α2. Moreover, the region between β4 and α2 is significantly expanded, as strand β4 shifts with no movement of the transmembrane α2 helix. These conformation changes in this region must unwind the ECH (Fig. 7c). Concomitantly, the extracellular part of helix α3 is wound up by about 20°, and thus D146 rotates, together with F147 and Y148, and forms a salt-bridge with R227′ of C-CPE (Fig. 7b). At the same time, L151 shifts by about 4 Å, and is inserted into the triple tyrosine pit of C-CPE. These conformation changes, coupled with the association between ECS2 and C-CPE, result in the movement of the V2 region, which shifts by as much as 7 Å to avoid steric hindrance with D225′ and S313′ of C-CPE (Fig. 7b).

**Figure 8:**
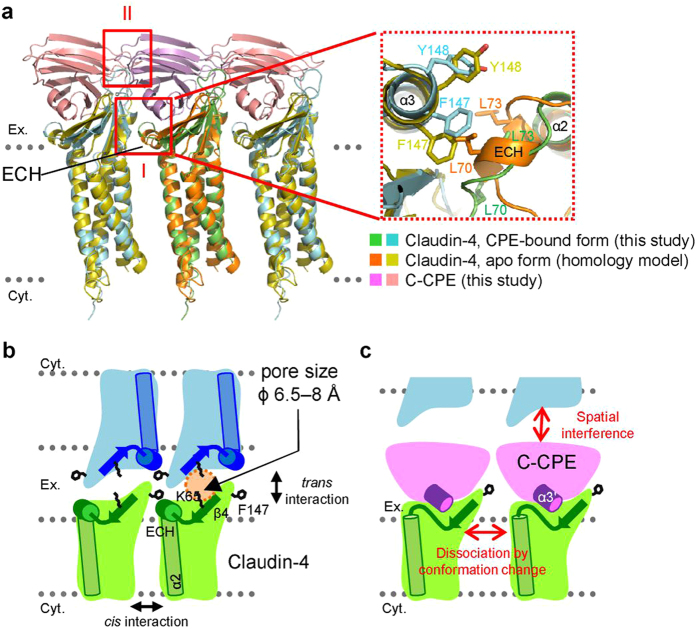
CPE disrupts claudin assembly in tight junctions. (**a**) C-CPE binding induces disruption of the *cis* interaction between claudin-4 molecules in the tight junction strand. Claudin-4 molecules in the C-CPE-complexed form and in the homology-modeled apo form, superimposed and aligned on the *b*-axis of the crystal packing of mouse claudin-15^13^. Box I indicates the major conformation changes resulting in the disruption of the *cis* interaction. Box I is rotated 90° upward and enlarged on the right. Box II indicates the putative collision between adjacent C-CPE molecules. (**b,c**) Models of the *trans* interaction between claudin-4 molecules in the tight junction strand (**b**) and the tight junction disruption by C-CPE binding (**c**).

What is the mechanism of tight-junction disruption triggered by C-CPE binding? It was proposed that the crystal packing of mouse claudin-15 along the *b* axis reflects the *cis* assembly of claudin-15 molecules with the tight-junction strand on the cell surface. This putative *cis* assembly is mediated by the interactions between the ECH of one molecule and the ECS2 of the next, or the intermolecular hydrophobic interactions between M68 (L70 in claudin-4) of the ECH region and F146 (F147) of the ECS2 region (box I in Fig. 8a)^13^. Our homology model of the apo-form and the crystal structure of the C-CPE-bound form of claudin-4 along the *b* axis of the mouse claudin-15 crystal are shown in Fig. 8a. The model indicated that the *cis* assembly of claudin-4 can be formed in the same manner as claudin-15. Together, these observations suggested that the conformation changes in the region around the ECH and V2 regions that occur upon CPE binding (Fig. 7a–c) disrupt the putative *cis* assembly of claudin-4 (box I, Fig. 8a).

Furthermore, if two C-CPE molecules bind to a pair of neighboring claudin-4 molecules arrayed in *cis*, then they would cause unavoidable steric hindrance (Fig. 8a, box II). On the basis of the *cis* assembly of claudin-4 (Fig. 8a), we propose a model of the tight-junction pore structure, including the possible *trans* assembly with C-CPE molecules on the opposite cell, as shown in Fig. 4b. In the case of claudin-2, a cysteine residue substituting for D65 (K65 on the palm side of the little finger of claudin-4) could form a disulfide bond within the *trans* assembly^21^. Therefore, it is highly likely that the 65^th^ position of one claudin-4 molecule is close to another claudin-4 molecule in the *trans* interaction (Fig. 8b). Although a pore (6.5–8 Å, ref. 22) exists between the palms of the *trans*-interacting claudin molecules, the present binding mode of C-CPE to the palm side of claudin-4 is not compatible with tight-junction formation, considering the size of C-CPE (approximately 40 × 25 × 25 Å^3^) and the full-length CPE (95 × 42 × 32 Å^3^)^17^ (Supplementary Fig. S7). This spatial competition for the claudin palm between the *trans* claudin molecule and the CPE might be one of the inhibition mechanisms (Fig. 8c).

The present novel findings on the mechanisms of tight-junction disruption might facilitate the development of anti-enterotoxin therapeutics. The previously reported C-CPE mutants that target other claudins were designed without any knowledge of the claudin structures^23^,^24^. This study now provides information about the claudin-4•C-CPE structure with the C-CPE-induced conformation changes of claudin-4. Therefore, the present complex structure is strongly expected to enable the rational design of novel C-CPE mutants that target other claudins, and innovative medically important technologies to regulate selectively, at will, the tight junctions formed by claudin family members in different organs.

## Methods

### Structure determination

The details of the cell-free synthesis of T4L-claudin-4 and C-CPE, the purification and crystallization of the T4L-claudin-4•C-CPE complex, and the X-ray diffraction data collection, data processing, and initial phasing have been described (Shinoda *et al*.^16^). The electron density maps corresponding to the α helices and the β strands of human claudin-4 were visualized by the molecular replacement (MR) – single-wavelength anomalous dispersion (SAD) method, using the program Phaser-EP^25^ in the PHENIX suite^26^, with the MR solution including four C-CPE molecules and two T4 lysozyme molecules. The Cα atoms of the α helices and the β strands of claudin-4 were traced, using the program Find Helices and Strands (PHENIX) with the density modified map. Using the MR-SAD method with the *P*4_3_2_1_2 crystal dataset which was collected using BL32XU at SPring-8^27^,^28^ and the preliminary model described above, the locations of 24 of the 30 selenium atoms were determined (Supplementary Fig. S8). On the basis of the positions of the identified selenium atoms, those of the other residues of claudin-4 were assigned. For the parts in which the residues could not be placed automatically, the amino acid residues were manually placed with the program COOT^29^. The atomic model was refined, using the restraints from the experimental phases, by repetitive model building and map calculations with the programs COOT and phenix.refine (PHENIX). NCS restraints were used in the refinement with the low resolution data, but not in the final refinement with the high resolution data. The quality of the model was examined with MolProbity^30^. The Ramachandran statistics of this model were 92.65% (favored), 7.30% (allowed), and 0.06% (outliers), and the clash score was 7.78. The *R*_work_/*R*_free_ values were 0.29/0.31. Structural graphics were drawn using PyMol (www.pymol.org) and UCSF Chimera^31^. The electrostatic potential on the surface of our structural model was calculated using the Adaptive Poisson-Boltzmann Solver^32^.

### Homology modeling

Homology modeling of the apo form of human claudin-4 was performed separately for the N-terminal part (M1–T34) and the C-terminal part (N42–S186) by SWISS-MODEL^33^, with the crystal structure model of mouse claudin-15 (ref. 13, PDB: 4P79) as the template. Helix α4 was not well modeled by SWISS-MODEL in the homology model of the claudin-4 apo form, possibly because its formation depends on the lipid bilayer environment. Therefore, the structure of α4 in mouse claudin-15 was retained, with manual replacements in the human claudin-4 sequence with COOT. The coordinate sets of the N- and C-terminal parts were merged, and refined by the program in CNS^34^ with no experimental energy term.

### Pull-down assay

The human claudin-4 (full-length) and the glutathione-*S*-transferase-tagged C-CPE (residues 185′–319′) (GST-C-CPE) used in this assay were both prepared by the *E. coli* cell-free protein synthesis method (Supplementary Fig. S9a–d). Approximately 10 μg of claudin-4 were incubated with 10 μg of GST-C-CPE at 4 °C for 1 hr with shaking, and then the solution was mixed with 20 μl of glutathione Sepharose resin (GE) at 4 °C for 2 hr with shaking. The resin was washed with 40 column volumes of 50 mM Tris-HCl buffer (pH 7.0), containing 0.05% β-dodecyl-*D*-maltopyranoside (βDDM, Anatrace), 0.002% cholesterylhemisuccinate (CHS, Anatrace), and 400 mM NaCl, and eluted with 50 μl of 50 mM Tris-HCl buffer (pH 8.0), containing 20 mM reduced glutathione, 0.05% βDDM, 0.002% CHS, and 400 mM NaCl. The eluates were analyzed by SDS-PAGE and CBB-staining (Supplementary Fig. S10). Densitometry of the bands in the SDS-PAGE gel was performed using ImageJ (http://imagej.nih.gov/ij/). The normalized bound values were calculated from the ratio of the density of the claudin band to the density of the corresponding C-CPE band, and normalized to the bound amount of the control sample (claudin-4 wild type and C-CPE wild type). Statistical analysis was performed using Welch’s t-test in Microsoft EXCEL.

## Additional Information

**Accession codes:** The Swiss-Prot accession codes for human claudin-4 and CPE are O14493 and Q0SVZ0, respectively. The coordinates and structure factors for the structure of the human claudin-4•C-CPE complex have been deposited in the Protein Data Bank, under the accession code 5B2G.

**How to cite this article**: Shinoda, T. *et al*. Structural basis for disruption of claudin assembly in tight junctions by an enterotoxin. *Sci. Rep.* **6**, 33632; doi: 10.1038/srep33632 (2016).

## Supplementary Material

Supplementary Information
