# Supplementary material for: Structural basis for disruption of claudin assembly in tight junctions by an enterotoxin
Source: Sci Rep. 2016 Sep 20;6:33632. doi: 10.1038/srep33632 (PMC5028891; doi:10.1038/srep33632)
Supplement: Supplementary Information [file srep33632-s1.pdf]

Supplementary Information

Structural basis for disruption of claudin assembly in tight junctions by an enterotoxin

Takehiro Shinoda[1,2], Naoko Shinya[1,2], Kaori Ito[1,2], Noboru Ohsawa[1,2], Takaho Terada[1,3], Kunio Hirata[4], Yoshiaki Kawano[4], Masaki Yamamoto[4], Tomomi Kimura-Someya[1,2], Shigeyuki Yokoyama[1,3]*, Mikako Shirouzu[1,2]*

[1]RIKEN Systems and Structural Biology Center, 1-7-22 Suehiro-cho, Tsurumi-ku, Yokohama 230-0045, Japan.

[2]Division of Structural and Synthetic Biology, RIKEN Center for Life Science Technologies, 1-7-22 Suehiro-cho, Tsurumi-ku, Yokohama 230-0045, Japan.

[3]RIKEN Structural Biology Laboratory, 1-7-22 Suehiro-cho, Tsurumi-ku, Yokohama 230-0045, Japan.

[4]RIKEN SPring-8 Center, 1-1-1, Kouto, Sayo-cho, Sayo-gun, Hyogo 679-5148, Japan.

*Correspondence to: E-mail: yokoyama@riken.jp (S.Y.); mikako.shirouzu@riken.jp (M.S.)

Supplementary Table S1 Data collection and refinement statistics

	Native T4L-claudin-4 •SeMet C-CPE (BL41XU)	Native T4L-claudin-4 •SeMet C-CPE (BL32XU[27, 28])	SeMet T4L-claudin-4 •SeMet C-CPE (BL32XU)
**Data collection**			
Space group	$P4_3$	$P4_3$	$P4_32_12$
No. crystals	1	1	1
Cell dimensions			
a, b, c (Å)	105.9, 105.9, 244.3	105.7, 105.7, 244.2	98.4, 98.4, 244.6
α, β, γ (°)	90, 90, 90	90, 90, 90	90, 90, 90
Resolution (Å)	50-3.35 (3.44-3.35)*	50-4.20 (4.31-4.20)	50-4.20 (4.45-4.19)
R_{meas}	0.161 (2.657)	0.201 (1.398)	0.215 (1.355)
$I/\sigma I$	6.63 (0.48)	6.90 (1.44)	8.49 (1.84)
Completeness (%)	99.6 (98.8)	99.8 (98.5)	99.7 (98.5)
Redundancy	4.55 (4.18)	6.05 (5.80)	8.37 (8.25)
CC^* **	0.999 (0.485)		
**Refinement**			
Resolution (Å)	48.59-3.50		
No. reflections	33738		
R_{work}/R_{free}	0.29 / 0.31		
No. atoms			
Protein	14200		
Ligand/ion	0		
Water	0		
B-factors			
Protein	99.91		
Ligand/ion	0		
Water	0		
R.m.s. deviations			
Bond lengths (Å)	0.005		
Bond angles (°)	1.172		

*Highest resolution shell is shown in parentheses.

**CC^* was calculated with the program Calculate CC^* in the PHENIX suite[26].

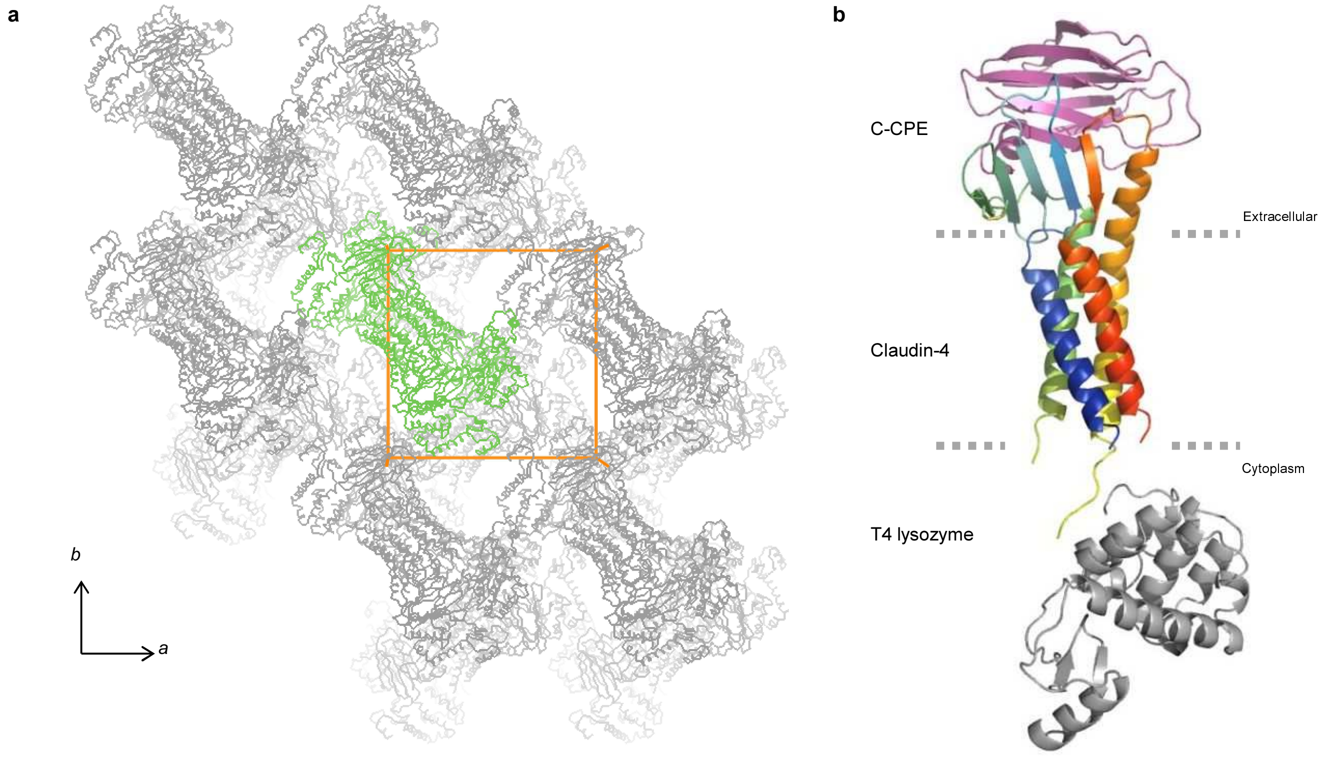

Supplementary Figure S1 | Crystal packing. (a) Crystal packing of the T4L-claudin-4•C-CPE complex in space group $P4_3$, as displayed on the *a*–*b* plane. (b) Overview of the T4L-claudin-4•C-CPE complex. Claudin-4 and C-CPE are colored as in Fig. 1, and T4L is colored gray.

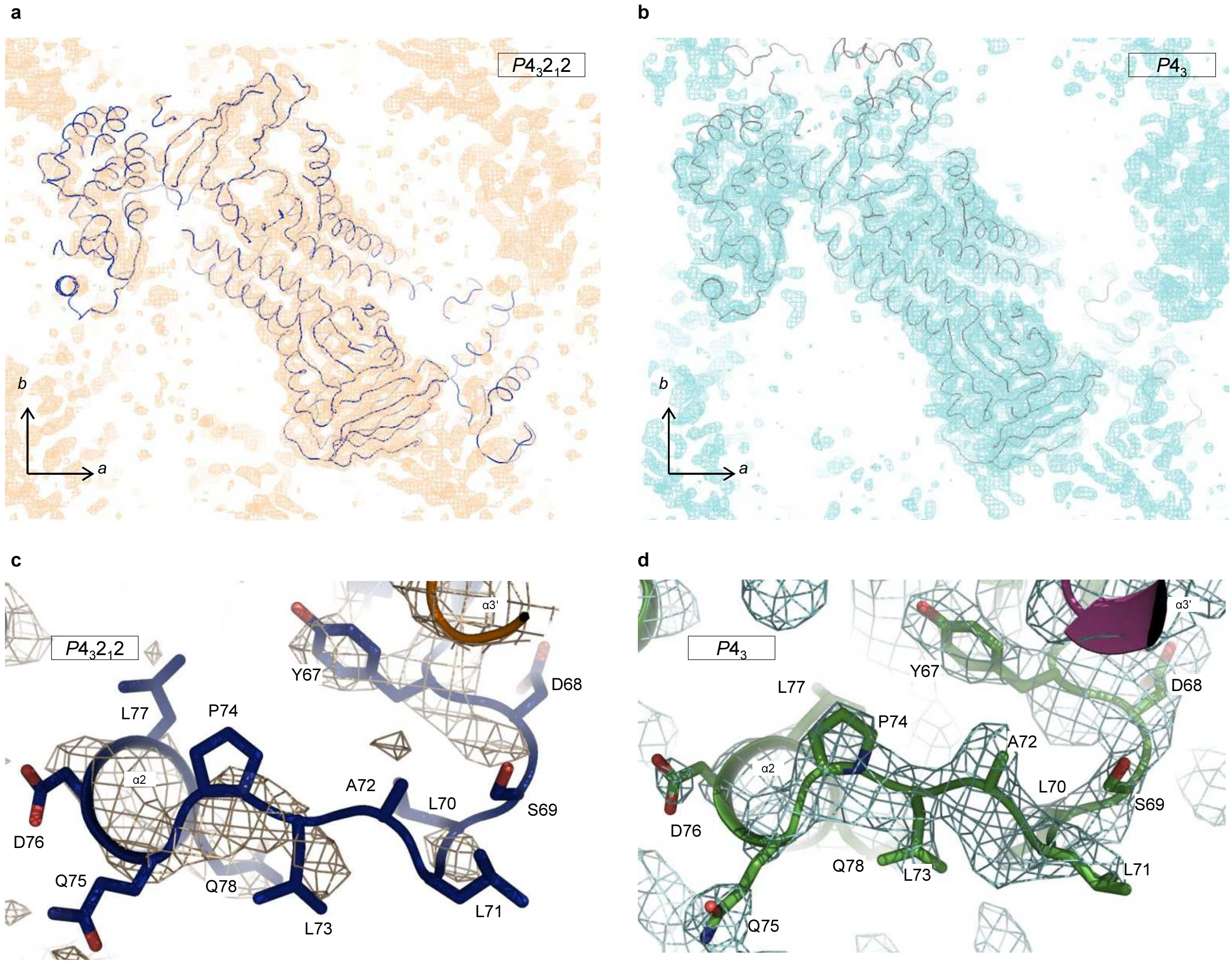

Supplementary Figure S2 | Comparison between the *P*4$_3$2$_1$2 crystal structure of the SeMet-T4L-claudin-4•SeMet-C-CPE complex and the *P*4$_3$ crystal of the T4L-claudin-4•C-CPE complex. (a, b) Overview of the asymmetric unit of the *P*4$_3$2$_1$2 crystal of the SeMet-T4L-claudin-4•SeMet-C-CPE complex (a) and the *P*4$_3$ crystal of the T4L-claudin-4•C-CPE complex (b), with 2*F*o-*F*c maps at 4.2 Å and 3.5 Å, respectively. (c, d) The β4–α2 loop in the *P*4$_3$2$_1$2 crystal (c) and the *P*4$_3$ crystal (d) with 2*F*o-*F*c map. The 2*F*o-*F*c maps are contoured at 1.5 σ.

a

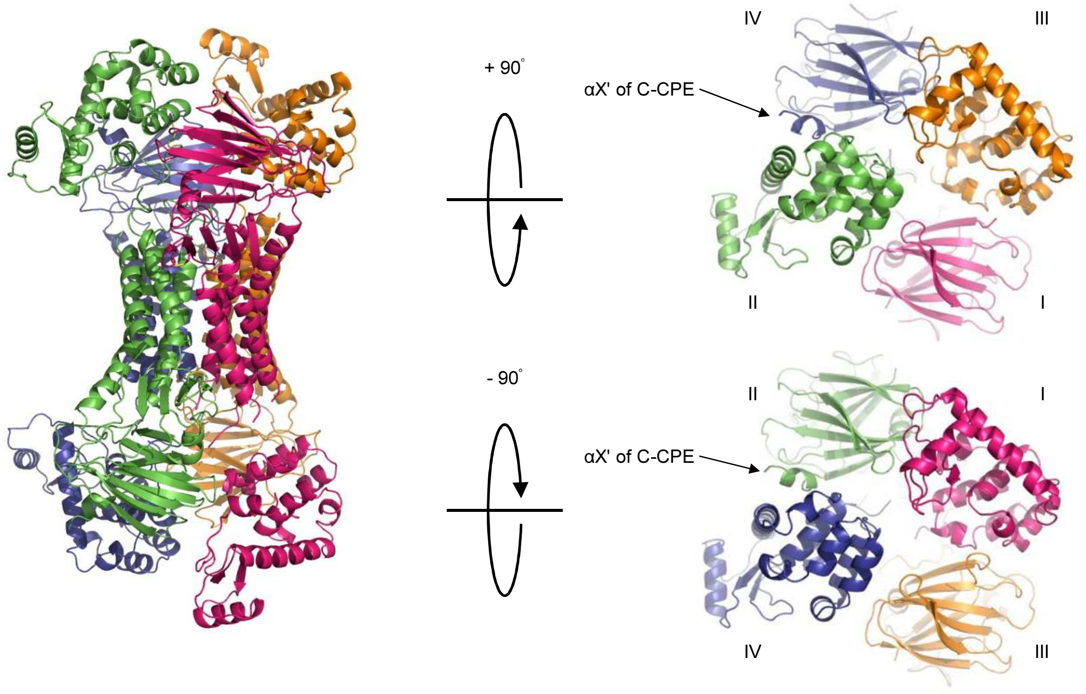

b

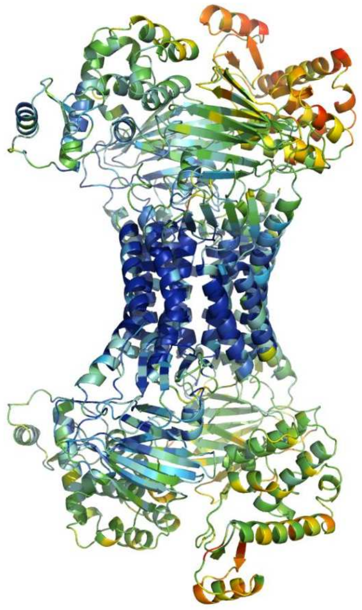

c

Complex	Modeled regions: T4 lysozyme	Modeled regions: Claudin-4	Modeled regions: C-CPE	Cα RMSD (Å): Claudin-4	Cα RMSD (Å): C-CPE	Cα RMSD (Å): C-CPE (vs 3AM2)
I	N1002–Y1161	G5–V100, D110–S184	A203–F319	–	–	0.53
II	G998–G1163	M1–S184	E196–F319	0.42	0.45	0.48
III	N1002–Y1161	M1–V100, D110–S184	I199–F319	0.31	0.37	0.54
IV	G998–G1163	M4–V99, K114–S184	E196–F319	0.44	0.47	0.48

Supplementary Figure S3 | Four complexes of T4L-human claudin-4•C-CPE in the asymmetric unit of the *P*4$_3$ crystal. (a) The asymmetric unit of the *P*4$_3$ crystal of the T4L-claudin-4•C-CPE complex contains four complexes: I (pink), II (green), III (orange), and IV (dark blue). (b) The temperature factors of the Cα atoms, ranging from 46 Å^2 (blue) to 207 Å^2 (red), on the four complexes in the asymmetric unit of the *P*4$_3$ crystal of the T4L-claudin-4•C-CPE complex. (c) Comparison of the structural models between the four independent complexes in the *P*4$_3$ crystal of the T4L-claudin-4•C-CPE complex. The root-mean-square deviations of the Cα atoms were calculated by the program PyMOL.

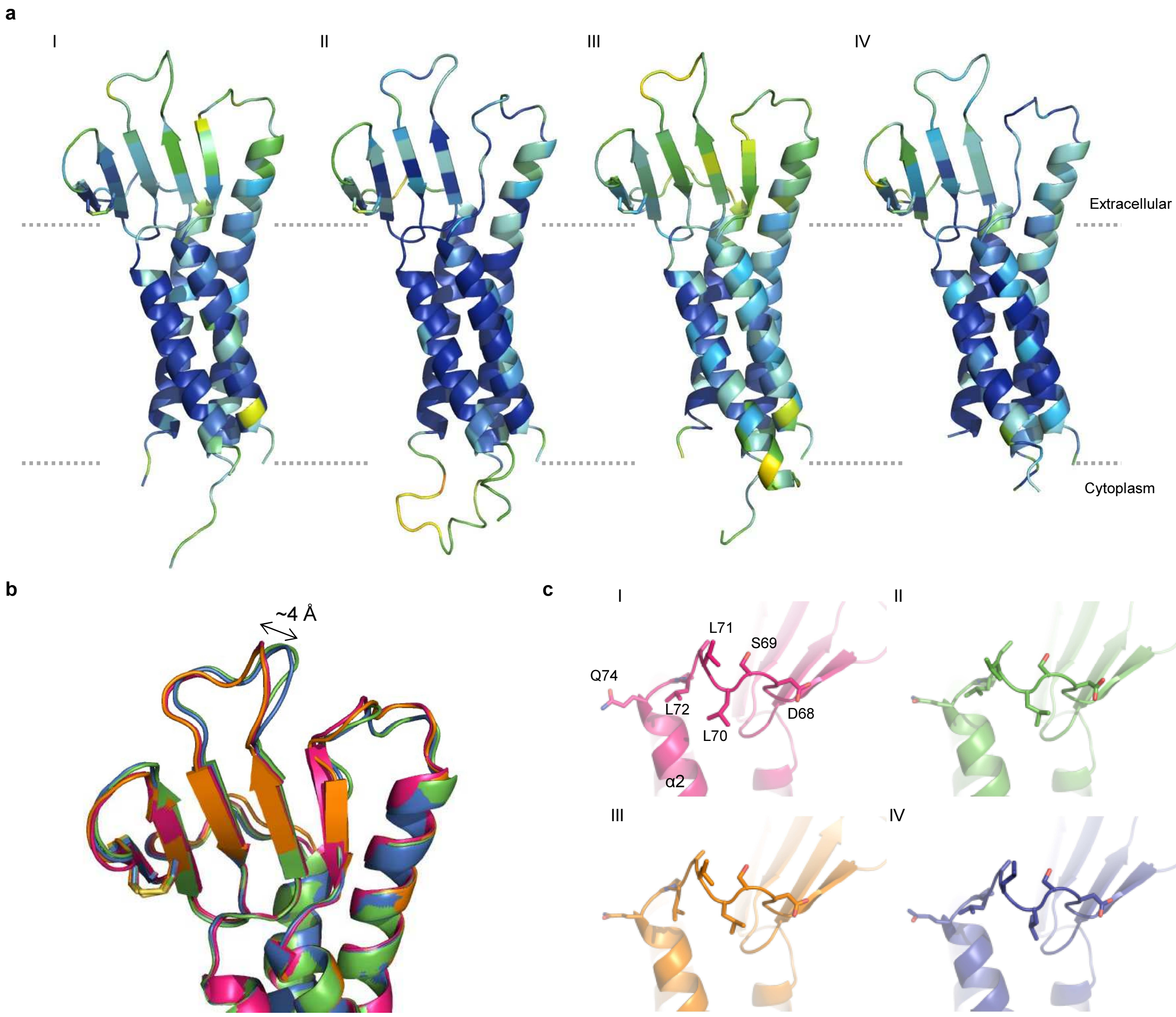

Supplementary Figure S4 | Comparison of the four structures of human claudin-4 in the asymmetric unit. (a) The overall structures of human claudin-4 from the four complexes with color-coded temperature factors of the Cα atoms, determined in the same manner as in Supplementary Fig. S3b. (b, c) Comparison of the extracellular region (b) and the β4-α2 loop (c) of each complex, colored in the same manner as in Supplementary Fig. S3a.

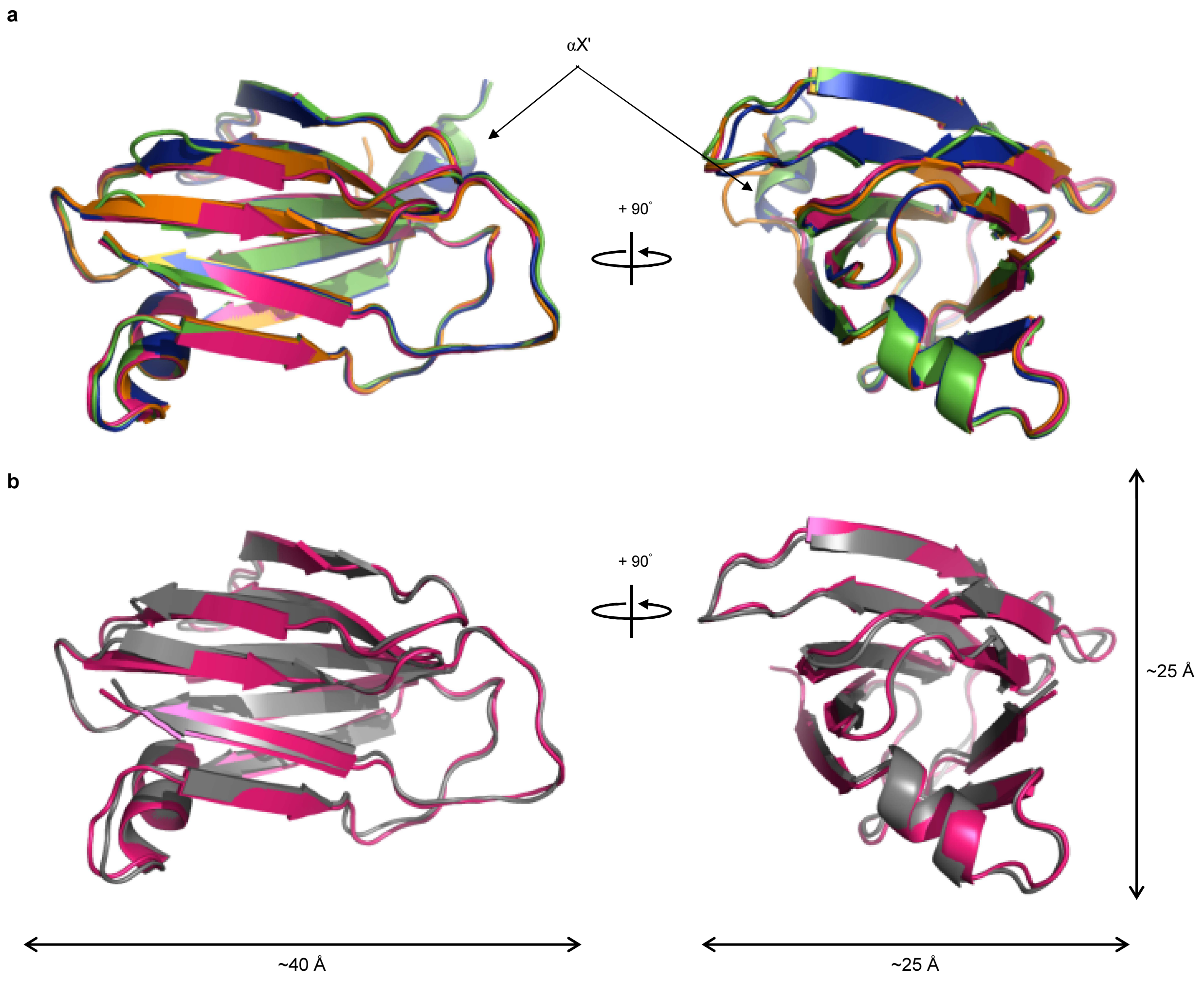

Supplementary Figure S5 | Comparison between the C-CPE structures. (a) Superimposition of the structures of the four independent C-CPE molecules, colored in the same manner as in Supplementary Fig. S3a, in the $P4_3$ crystal of the T4L-claudin-4•C-CPE complex. (b) Superimposition of the structure of C-CPE in complex I with T4L-claudin-4 (pink) on that of C-CPE (residues 205–319) in the full-length, free form of CPE (PDB: 3AM2) [17] (gray).

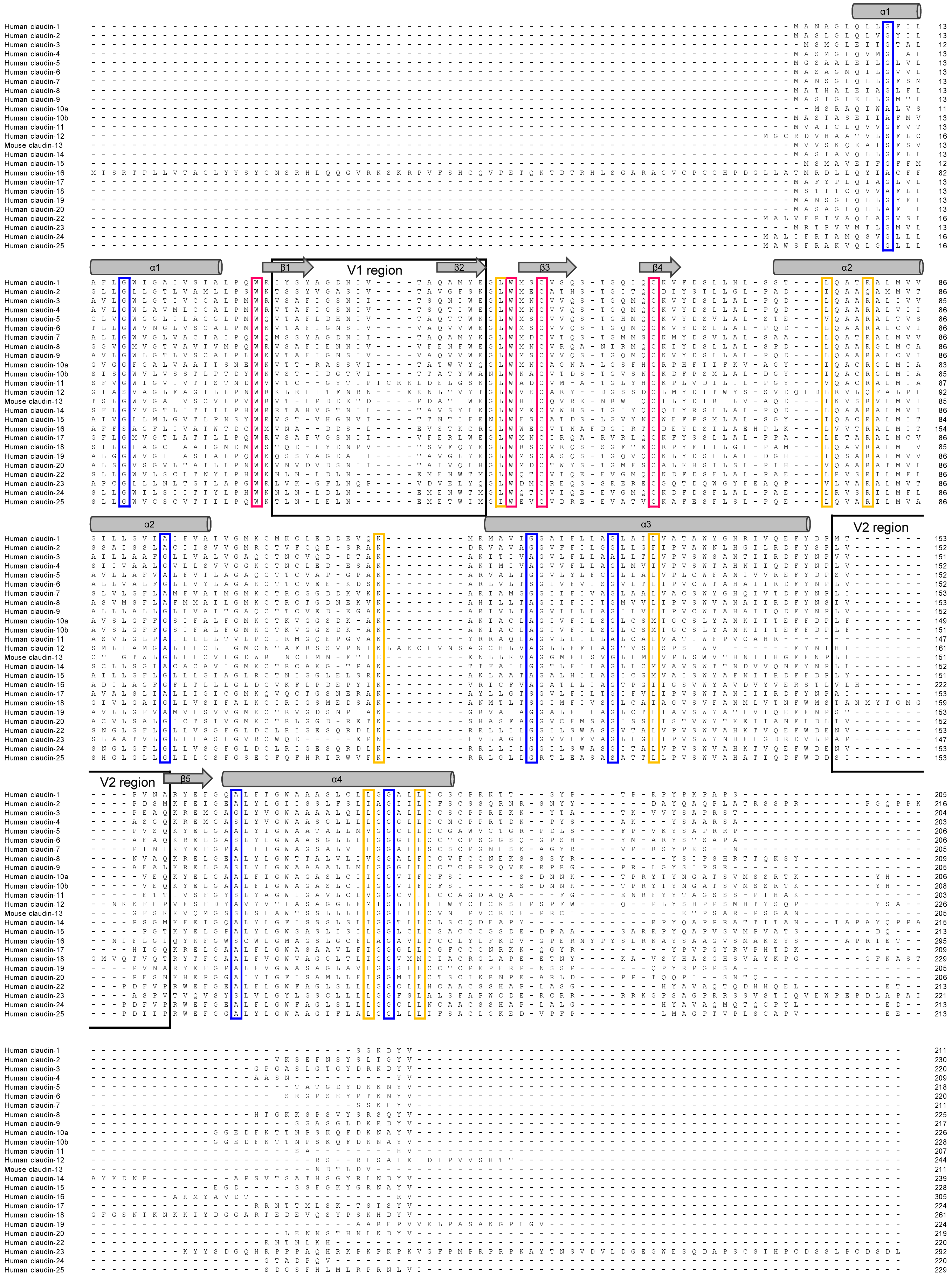
α1
V1 region
β1
β2
β3
β4
α2
α3
V2 region
β5
α4

Supplementary Figure S6 | Sequence alignment of human claudins. The identical residues, highly conserved residues, and homologous residues are boxed in red, orange and blue, respectively. The alignment was generated with Clustal Omega. The secondary structure elements of human claudin-4 are indicated above the sequences.

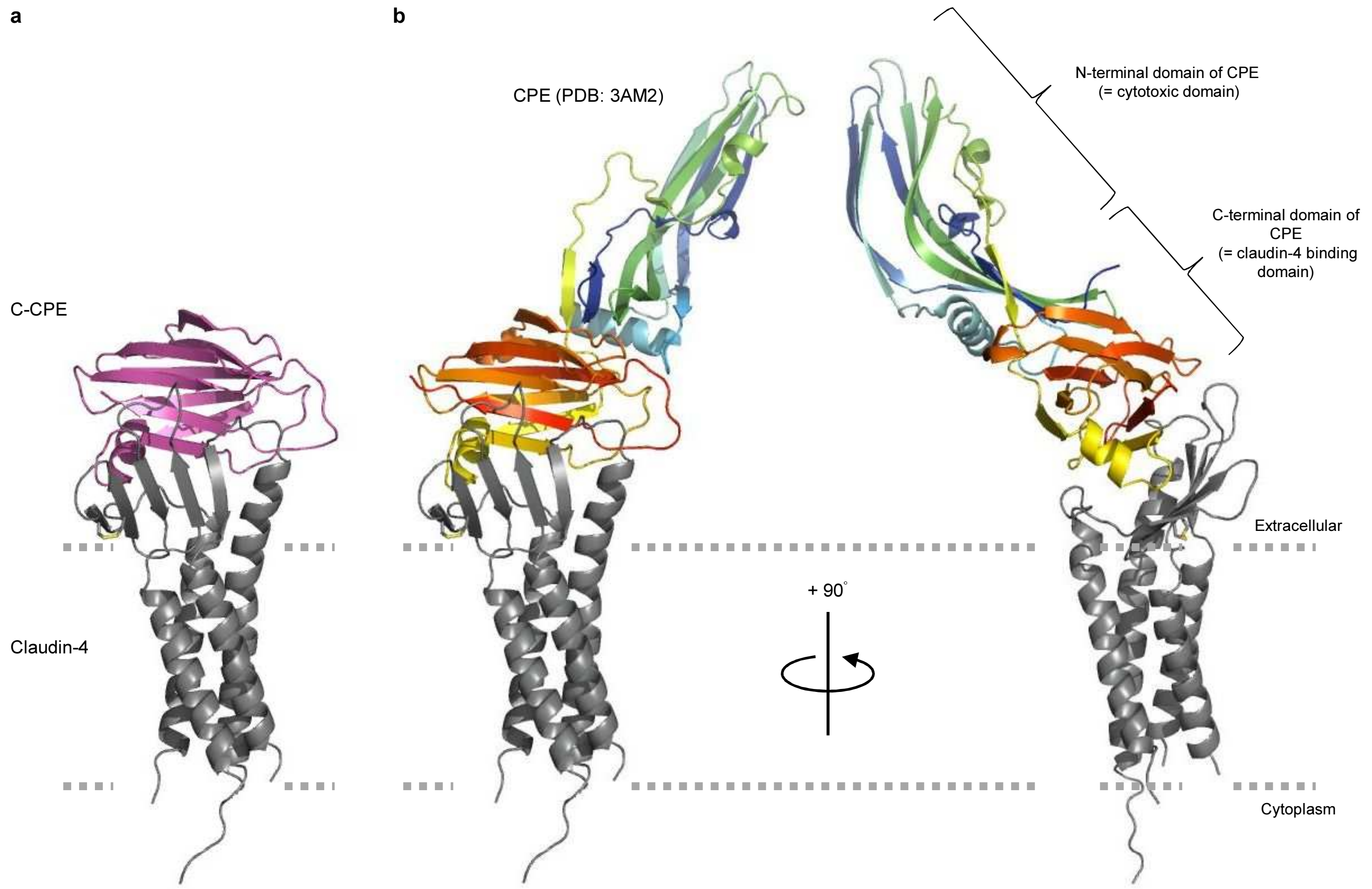

Supplementary Figure S7 | A model of the human claudin-4•full-length CPE complex. (a) The human claudin-4•C-CPE complex determined in this study. Human claudin-4 and C-CPE are colored gray and purple, respectively. (b) The model of the human claudin-4•full-length CPE complex, generated by the superimposition of the full-length CPE (PDB: 3AM2, ref.17) onto the C-CPE in the present structure. The full-length CPE is rainbow colored.

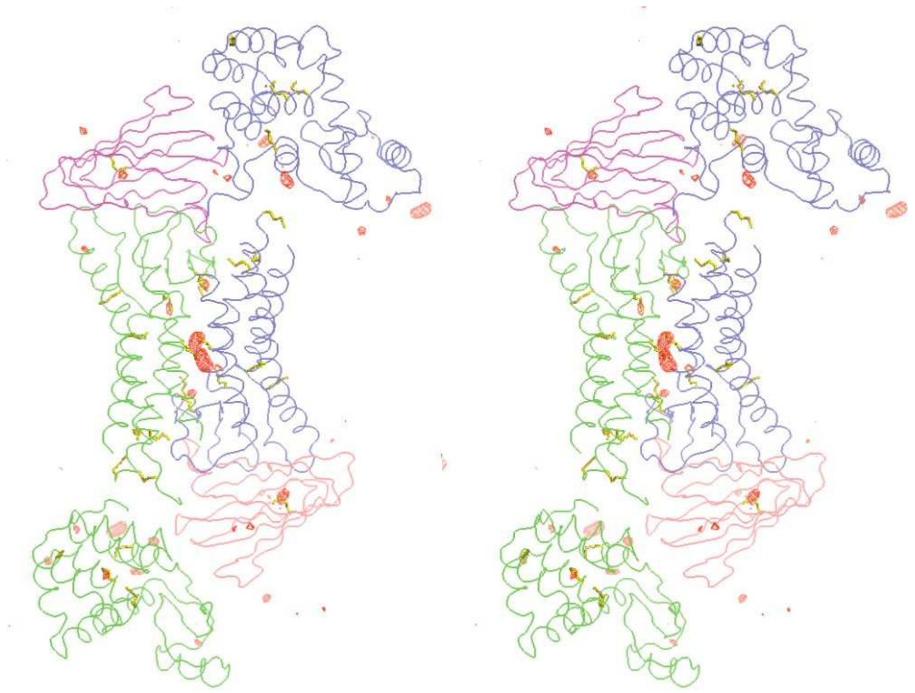

Supplementary Figure S8 | Selenomethionine positions in the *P*$4_3 2_1 2$ crystal structure of the SeMet-T4L-claudin-4•SeMet-C-CPE complex. The asymmetric unit of the *P*$4_3 2_1 2$ crystal, composed of two SeMet-T4L-claudin-4•SeMet-C-CPE complexes, is represented in a stereoview. In complex I, T4L-claudin-4 and C-CPE are colored green and purple, respectively. In complex II, T4L-claudin-4 and C-CPE are colored blue and pink, respectively. Anomalous difference densities, contoured at 4 σ, are shown in red. Selenomethionines are represented by yellow sticks.

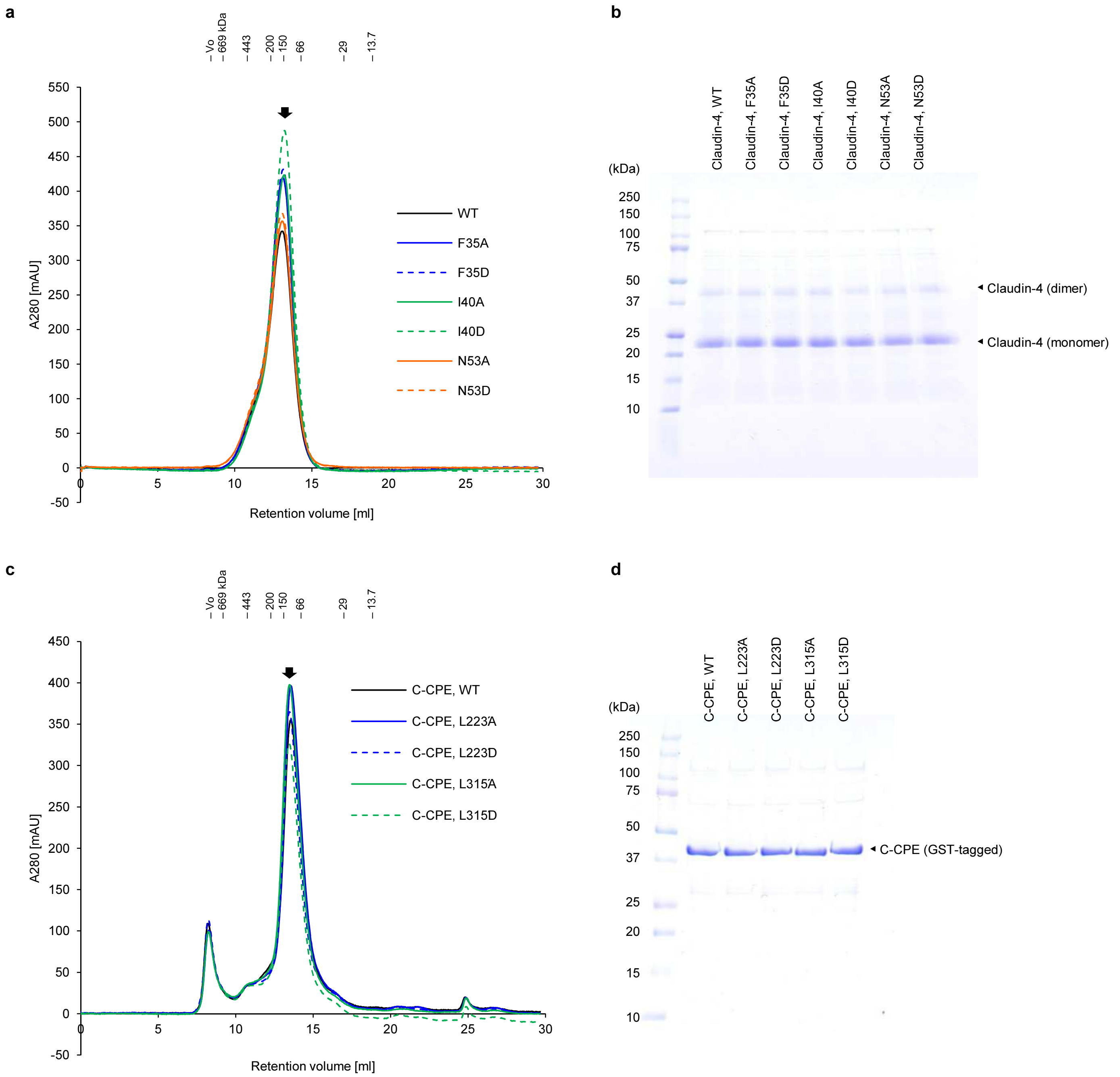

Supplementary Figure S9 | Size-exclusion chromatography analysis of the claudin-4 mutants and the C-CPE mutants that appear in Fig. 4i-j. (a, c) The Superdex 200 10/300 column (GE Healthcare) elution profiles of (a) the human claudin-4 mutants and (c) the GST-tagged C-CPE mutants. The protein samples were produced by the *E. coli* cell-free protein synthesis method, and purified by tag-affinity chromatography with the N11 tag at the N-terminus of the proteins. (b, d) The SDS-PAGE gel images of the peak fractions indicated by the black arrows in (a) and (c). In (b) and (d), 0.9 µg and 0.5 µg of protein were fractionated in each lane. SDS-PAGE gels were stained with Coomassie Brilliant Blue.

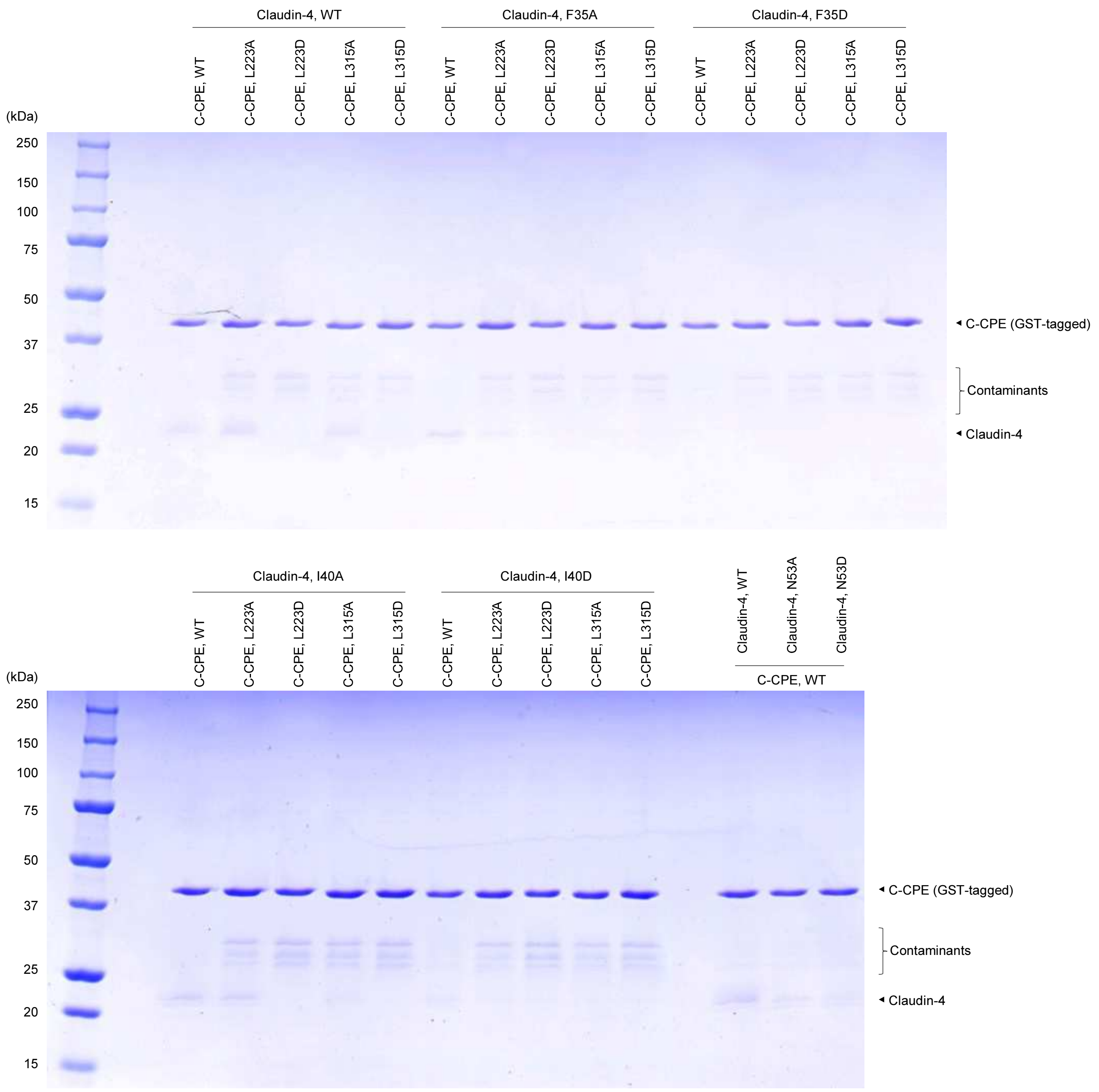

Supplementary Figure S10 | SDS-PAGE gel images of the pull-down assay in Fig. 4i-j. An equal volume of each eluate was applied to the well, and fractionated by SDS-PAGE with a 10–20% gradient gel. SDS-PAGE gels were stained with Coomassie Brilliant Blue.
